# Celastrol Stabilizes Glycolipid Metabolism in Hepatic Steatosis by Binding and Regulating the Peroxisome Proliferator-Activated Receptor γ Signaling Pathway

**DOI:** 10.3390/metabo14010064

**Published:** 2024-01-19

**Authors:** Mingzhu Luo, Yiting Wang, Yanyan Ma, Jingzhe Li, Jingyi Wang, Changzhen Liu

**Affiliations:** Experimental Research Center, China Academy of Chinese Medical Sciences, Beijing 100700, China; luomingzhu@merc.ac.cn (M.L.); wangyiting@merc.ac.cn (Y.W.); mayanyan@merc.ac.cn (Y.M.); lijingzhe@merc.ac.cn (J.L.); wangjingyi@merc.ac.cn (J.W.)

**Keywords:** celastrol, PPARγ, hepatic steatosis, adipocyte differentiation, SPR

## Abstract

The prevalence of nonalcoholic fatty liver disease (NAFLD) has been increasing. Obesity, insulin resistance, and lipid metabolic dysfunction are always accompanied by NAFLD. Celastrol modulates the Peroxisome proliferator-activated receptor γ (PPARγ) and CCAAT/enhancer binding protein α (C/EBPα) signaling pathways, thereby promoting lipolysis in 3T3-L1 adipocytes. In the present study, oleic-acid-induced NAFLD and differentiated 3T3-L1 preadipocytes were used as models of NAFLD and obesity to investigate the protective effect of celastrol. We investigated the impact of celastrol on hepatic steatosis caused by oleic acid (OA), as well as the associated underlying molecular pathways. To address the aforementioned questions, we used a cellular approach to analyze the signaling effects of celastrol on various aspects. These factors include the improvement in fatty liver in HepG2 cells, the differentiation of 3T3-L1 preadipocytes, glucose uptake, and the modulation of key transcriptional pathways associated with PPARγ. The administration of celastrol effectively mitigated lipid accumulation caused by OA in HepG2 cells, thereby ameliorating fatty liver conditions. Furthermore, celastrol suppressed the impacts on adipocyte differentiation in 3T3-L1 adipocytes. Additionally, celastrol exhibited the ability to bind to PPARγ and modulate its transcriptional activity. Notably, the ameliorative effects of celastrol on hepatic steatosis were reversed by rosiglitazone. According to our preliminary findings from in vitro celastrol signaling studies, PPARγ is likely to be the direct target of celastrol in regulating hepatic steatosis in HepG2 cells and adipocyte differentiation in 3T3-L1 cells.

## 1. Introduction

Nonalcoholic fatty liver disease (NAFLD), renamed as metabolic-associated fatty liver disease (MAFLD), is widely acknowledged as the hepatic manifestation of metabolic syndrome and encompasses NAFLD and nonalcoholic steatohepatitis [1,2,3]. An abnormal lipid buildup in the liver, often accompanied by obesity, type 2 diabetes, and metabolic dysfunction in the absence of alcohol abuse, defines NAFLD [4,5]. Considering its global prevalence and its correlation with numerous life-threatening chronic ailments, NAFLD poses a significant health concern [6,7]. The control of the makeup and buildup of lipids in the liver involves an intricate web of interconnected metabolic routes, encompassing lipid production, breakdown, β-oxidation, storage, and transportation. The disruption of any of these pathways can cause the liver to accumulate lipids, resulting in hepatic steatosis [8,9]. Peroxisome proliferator-activated receptor γ (PPARγ) is a nuclear receptor that plays a central role in maintaining lipid and glucose balance. PPARγ can be activated by various ligands, including both endogenous and exogenous molecules, such as fatty acids and rosiglitazone. Previous studies [10] have demonstrated an upregulation of PPARγ2 in NAFLD. Additionally, an overexpression of PPARγ can result in liver steatosis, a condition that can be mitigated through the administration of GW9662 [11]. In addition, it has been observed that the PPARγ inhibitor GW9662 can ameliorate high-fat-diet-induced fatty liver and insulin resistance [12]. Conversely, the administration of rosiglitazone exacerbates the syndrome associated with high-fat-diet-induced NAFLD [12].

Celastrol is derived from certain plants of the Celastraceae family, including *Tripterygium wilfordii* and *Celastrus orbiculatus*. Celastrol has been found to possess anti-obesity properties as reported in 2015 [13,14]. The administration of celastrol resulted in an augmentation of lipolysis and hindered the differentiation of adipocytes into 3T3-L1 adipocytes through the regulation of the PPARγ2 and CCAAT/enhancer binding protein (C/EBPα) signaling pathways [7,14]. Celastrol has the potential to mitigate hepatic lipotoxic injury by stabilizing the function of glucolipid metabolism [15,16]. Celastrol decreases lipid synthesis and enhances the antioxidative and anti-inflammatory status in NAFLD by upregulating Silent mating type information regulation 2 homolog 1 (Sirt1) [17]. Sirt1 enhances fatty acid mobilization from the adipose tissue and hepatic fatty acid oxidation by inhibiting PPARγ [18]. However, whether celastrol can improve NAFLD in vitro and whether it can control fatty liver disease and overweight by modulating the PPARγ signaling pathway need to be clarified. Although celastrol shows potential as a viable option for improving leptin sensitivity and combating obesity, additional research is needed to clarify its precise impact and the underlying mechanism in NAFLD. Oleic acid (OA)-induced NAFLD and differentiated 3T3-L1 preadipocytes are used in cellular studies of NAFLD and obesity. We conducted cellular signaling studies to elucidate the impact and potential mechanism of action of celastrol in NAFLD induced by OA in HepG2 cells, as well as its underlying interaction with lipolysis and adipogenesis in 3T3-L1 adipocytes.

## 2. Materials and Methods

### 2.1. Chemicals

The chemical agents used were as follows: celastrol (MedChenExpress, Monmouth Junction, NJ, USA), rosiglitazone (Selleck, Houston, TX, USA), GW9662 (Selleck, Houston, TX, USA), insulin-D-10 and 3-isobutyl-1-methylxanthine (IBMX), dimethyl sulfoxide (DMSO) (Sigma, St. Louis, MO, USA), and dexamethasone (DEX) (MedChenExpress, Monmouth Junction, NJ, USA). 2-NBDG was obtained from Caymen Chemical (An Arbor, MI, USA).

### 2.2. Culture and Steatosis of HepG2 Cells

HepG2 cells were cultured in Dulbecco’s Modification of Eagle’s Medium (DMEM) supplemented with 10% fetal bovine serum (FBS) (Gibco, Baltimore, MD, USA) at 37 °C in a 5% CO_2_ atmosphere. After reaching approximately 45% confluence, the cells were subjected to treatment with 1 mM OA bovine complex for another 48 h. Subsequently, the cells were treated with 1 µM rosiglitazone, 5 µM GW9662, or 0.5 µM celastrol for a duration of 48 h prior to conducting Oil Red O staining. The cells in each group had 3 complex holes, and the methods of observation and analysis were the same as those in the above experiments. The HepG2 cells are obtained from Experimental Research Center China Academy of Chinese Medical Sciences. The method establishment of OA-induced hepatic steatosis cells is shown in Figure S1.

### 2.3. Adipocytic Differentiation of 3T3-L1 Cells

The culturing of 3T3-L1 preadipocytes was performed using complete media supplemented with 10% heat-inactivated newborn calf serum (NBCS) (Gibco). The culture medium was supplemented with lipogenic agents (0.5 mM IBMX, 1 µM DEX, 2 µM insulin, and 0.5 µM rosiglitazone with 10% heat-inactivated FBS) to induce adipocytic differentiation after 48 h of confluence. Afterward, 3T3-L1 adipocytes were exposed to 2 µM insulin and pretreated with 1 µM rosiglitazone, 5 µM GW9663, or 0.5 µM celastrol for a duration of 48 h. After removing the drug, the cells were washed and cultured in drug-free medium supplemented with 2 µM insulin for 2–4 days. Each of the treatments was performed in triplicate. The cells were subjected to Oil Red O (ORO) staining on days 6–8 of differentiation. The glucose consumption and triglyceride (TG) levels in the culture supernatants were detected. The 3T3-L1 preadipocytes are obtained from Experimental Research Center China Academy of Chinese Medical Sciences.

### 2.4. Cell Viability Assay

After undergoing adipocyte differentiation, mature 3T3-L1 adipocytes were exposed to rosiglitazone, GW9662, and varying concentrations (0, 0.2, 0.5, 1 µM) of celastrol for 48 h. Cells incubated with 0.1% DMSO were utilized as control samples. Each concentration was repeated six times. After treatment, the cells were subjected to the Cell Counting Kit-8 (CCK8) (Dojindo, Tokyo, Japan) assay, and the absorbance was quantified at 450 nm using a microplate reader.

### 2.5. Oil Red O Staining

Initially, the cells were washed twice with Phosphate-Buffered Saline (PBS) and then subsequently fixed with 4% paraformaldehyde for 10 min at room temperature. Following fixation, the cells were rinsed three times with ddH_2_O. The cells were then treated with a homogeneous mixture of ORO staining solution and ddH_2_O at a ratio of 6:4 for 35 min. Subsequently, the cells were rinsed thrice with ddH_2_O. Using an inverted microscope, the visualization of the stained lipid droplets was visualized. To determine the amount of lipids inside the cells, the ORO that had attached to the cells was extracted using 70% isopropanol. The absorbance was measured in triplicate wells at 500 nm using a microplate reader.

### 2.6. PPARγ Agonist Reverse Experiment

After reaching approximately 45% confluence, the HepG2 cells were subjected to treatment with 5 µM GW9662, 0.5 µM celastrol, or an equivalent amount of solvent. Simultaneously, all the groups were administered 1 µM rosiglitazone for 48 h. Subsequently, the sections were treated with 1 mM OA bovine complex for another 48 h prior to conducting the ORO staining. Each of the treatments was performed in triplicate.

### 2.7. Glucose Absorption Assay

Human hepatocellular carcinoma HepG2 cells were chosen because they are a commonly used cell line in the literature for liver disease. HepG2 cells were seeded in 6-well plates at 7 × 10^4^ cells per well and exposed to DMSO, rosiglitazone, GW9662, or celastrol for 48 h. Each of the treatments was performed in triplicate. The cells were activated using 10 µg/mL insulin-D-10. Subsequently, the culture medium was incubated at 37 °C with 5% CO_2_ for 30 min. Finally, glucose uptake was measured and analyzed using flow cytometry. 2NBDG absorption was stopped by eliminating the incubation solution and performing two rinses with prechilled PBS. Afterward, the cells in each well were subsequently resuspended in 200 µL of PBS and then analyzed via flow cytometry for a period of 30 min.

### 2.8. Glucose Consumption Assay

The culture supernatants were subjected to glucose quantification. The glucose consumption levels were determined using a glucose content detection kit (Jiancheng, Nanjing, China, A154) following the guidelines provided by the manufacturer.

### 2.9. Triglyceride Content Assay

The protein concentration was determined using the bicinchoninic acid (BCA) protein assay (Thermo, Waltham, MA, USA). The levels of TG release levels were determined by employing a TG content detection kit (Jiancheng, Nanjing, China, A110).

### 2.10. Quantitative Real-Time Polymerase Chain Reaction (qRT-PCR)

The cells were collected, rapidly frozen, and maintained at a temperature of −80 °C until they were ready for additional procedures. Total RNA was isolated using the TRIzol reagent. Subsequently, the RNA was converted into complementary DNA (cDNA) by utilizing the Toyobo reverse-transcription kit (Toyobo, Osaka, Japan). The quantification of mRNA expression levels was performed via qRT-PCR analysis with a StepOne Real-Time PCR System (ABI, Los Angeles, CA, USA). The sequences of primers used in this study can be found in Table 1. The expression of the target mRNA in each sample was standardized in relation to the mRNA expression of the housekeeping gene, glyceraldehyde-3-phosphate dehydrogenase (GAPDH). Relative mRNA expression levels were calculated using the 2^−ΔΔCt^ approach.

### 2.11. Ligand Binding Assay

The binding affinity between the PPARγ protein and drugs was determined using a surface plasmon resonance (SPR)-based ligand binding assay, following the manufacturer’s protocol. First, the PPARγ protein was linked to a CM5 chip through the amine conjugation technique. After the compounds were dissolved in DMSO, they were diluted consecutively using a running buffer (HBS-EP; 0.01 M HEPES (pH 7.4), 0.15 M NaCl, 3 mM EDTA, and 0.005% (*v*/*v*) surfactant P20). Afterward, the diluted compounds were injected into both the PPARγ-coupled and reference channels. The durations for binding and dissociation were established as 60 s and 180 s, correspondingly.

An indirect ligand binding assay (unpublished) was constructed, and this method demonstrated the relationship between celastrol and PPARγ by introducing steroid receptor coactivator (SRC) 1. SRC1, a PPARγ coactivator, can be captured by PPARγ when agonists bind PPARγ. After compound loading, the response value of SRC1 to PPARγ changes with the properties of the compound. Rosiglitazone, a full agonist, served as a positive control, and buffer was used as a blank control.

### 2.12. Statistical Analysis

All results are presented as the mean ± standard error (SEM). Multigroup comparisons were conducted through one-way analysis of variance followed by Dunnett’s test. Statistical analysis was performed using GraphPad Prism software (version 7.0; GraphPad Software, Inc., San Diego, CA, USA). Statistical significance was determined at a threshold of *p* < 0.05.

## 3. Results

### 3.1. Celastrol Protects against Oleic-Acid-Induced Fatty Liver

**Figure 1:**
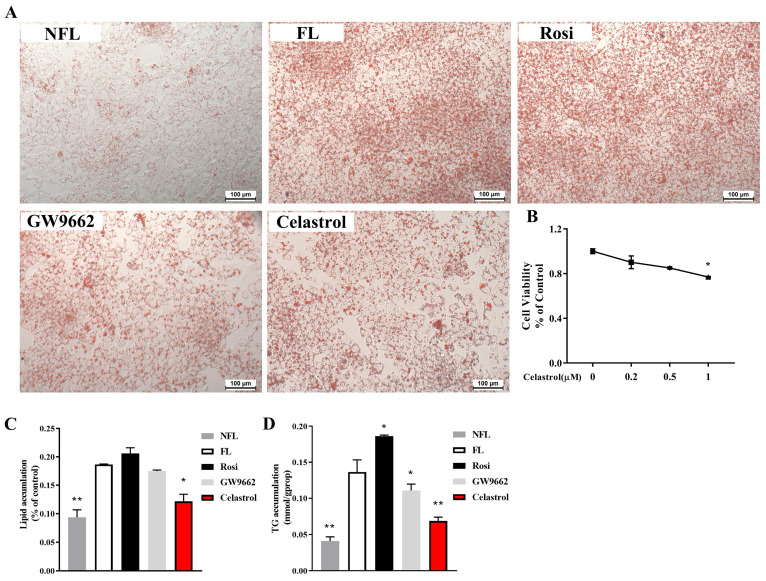
Celastrol inhibits OA-induced hepatic steatosis in HepG2 cells. OA-induced hepatic steatosis in HepG2 cells was treated with celastrol at 0.5 µM for 48 h and lipid accumulation was measured by (**A**) ORO staining and quantified by (**C**) biochemical analysis and (**D**) densitometry analysis. (**B**) Viability of HepG2 cells treated with celastrol at concentrations of 0, 0.2, 0.5, and 1 µM for 48 h. All the data were analyzed using one-way ANOVA followed by a post hoc test for multiple comparisons. The values are presented as the means ± SEMs; n = 3; * *p* < 0.05, ** *p* < 0.01 vs. the FL group. OA: oleic acid; ORO: Oil Red O; NFL: nonfatty liver group; FL: fatty liver group; Rosi: rosiglitazone.

HepG2 cells were treated with various concentrations of celastrol (0, 0.2, 0.5, 1 µM), and cell viability was measured via a CCK8 assay. According to Figure 1B, the findings indicated that cell viability was not affected by 0.2 µM and 0.5 µM celastrol, demonstrating no evidence of toxicity. Thus, we used a nontoxic concentration of 0.5 µM of celastrol in subsequent experiments.

In obese mice, celastrol, a potent antiobesity agent, can suppress food consumption, block the reduction in energy expenditure, and result in a weight loss of up to 45% [13]. NAFLD often occurs due to hepatic lipid accumulation, which is commonly linked to obesity. To examine the impact of celastrol on fatty liver disease, HepG2 fatty cells were induced with 1 mM OA and subsequently subjected to 48 h of treatment with celastrol. Celastrol improved the lipid accumulation in HepG2 cells with fatty liver induced by OA, as shown by the ORO staining and quantification (Figure 1A,C). TG levels were significantly increased by rosiglitazone and downregulated by celastrol and GW9662 (Figure 1D).

### 3.2. Celastrol Directly Binds PPARγ and Inhibits Its Transcriptional Activity

**Figure 2:**
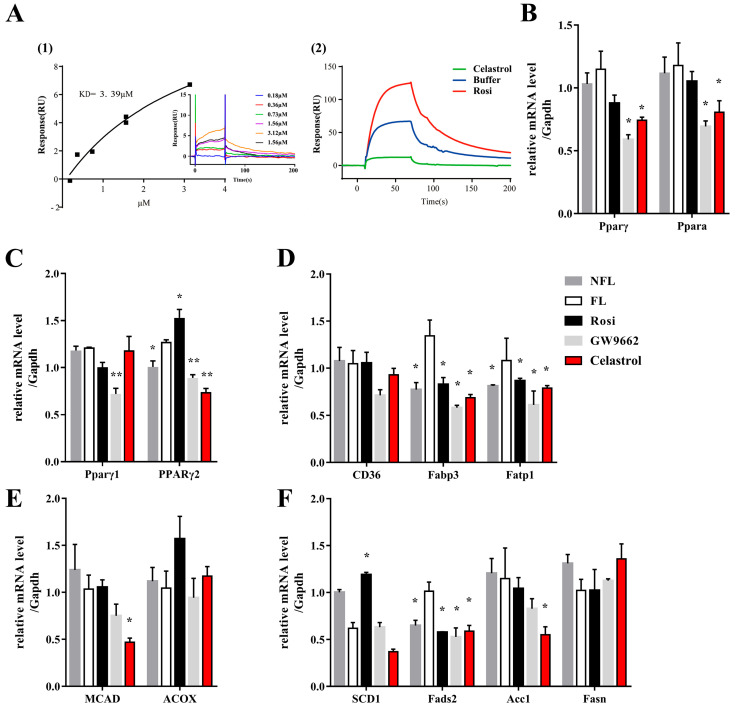
Celastrol can directly inhibit PPARγ transcriptional activity. (**A1**) The affinity fitting curve and affinity value (KD) generated by the binding of celastrol to PPARγ at different concentrations. (**A2**) The response value of SRC1 generated by the binding of celastrol to PPARγ determined via an indirect ligand binding assay. The OA-induced hepatic steatosis of HepG2 cells was induced by treatment with 0.5 µM celastrol for 48 h, and the mRNA levels of (**B**) PPARγ and PPARα, (**C**) PPARγ 1 and 2, (**D**) CD36, Fabp3, and Fatp1, (**E**) MCAD and ACOX, and (**F**) SCD1, Fads2, Acc1, and Fasn were measured. The values are presented as the means ± SEMs; n = 3; * *p* < 0.05, ** *p* < 0.01 vs. the FL group. OA: oleic acid; NFL: nonfatty liver group; FL: fatty liver group; Rosi: rosiglitazone; KD: affinity value.

Since the levels of lipids and glucose were modulated by celastrol, and since the PPARγ signaling pathway participates in the metabolism of glucose and lipids [19], the effect of celastrol on PPARγ was investigated. Direct methods based on SPR technology for ligand binding detection were employed. Celastrol was diluted with running buffer to a series of concentrations (0.18, 0.36, 0.73, 1.56, and 3.12 µM), and each concentration of celastrol was added to the surface of the coupled PPARγ-ligand binding domain (LBD) protein chip to achieve full binding between celastrol and the protein. Celastrol can bind to PPARγ-LBD (Figure 2(A1)), and the value of the equilibrium dissociation constant (KD) between celastrol and PPARγ-LBD was 3.39 µM. These results revealed that celastrol can bind PPARγ and that the ability of celastrol to improve OA-induced hepatic steatosis might be due to the PPARγ signaling pathway. An indirect ligand binding assay showed that rosiglitazone increased the capture of SRC1 by PPARγ, whereas celastrol decreased this capture (Figure 2(A2)). SRC was recruited by PPARγ and activated PPARγ-mediated gene transcription. Thus, these results indicated that celastrol might be an inhibitor of PPARγ and inhibit its transcriptional activity.

We hypothesized that the lipid-lowering efficacy of celastrol was a result of the altered transcriptional activity of PPARγ, although the lipid-lowering effects of different PPARγ ligands do not correspond with changes in the expression of genes regulated by PPARγ [20]. After OA-induced hepatic steatosis, we evaluated the expression of PPARγ and its downstream genes, including genes involved in lipid transport, synthesis, and β-oxidation, following treatment with celastrol, rosiglitazone, or GW9662. Celastrol and GW9662, two kinds of PPARγ inhibitors, both decreased the mRNA expressions of PPARγ and Peroxisome proliferator-activated receptor α (PPARα) (Figure 2B). Celastrol improves lipid synthesis by regulating the PPARγ signaling pathway. PPARγ2 was upregulated in NAFLD patients. In the present study, celastrol downregulated the mRNA levels of PPARγ2, and GW9662 decreased both the PPARγ1 and 2 mRNA levels (Figure 2C). Moreover, rosiglitazone upregulated the mRNA level of PPARγ2. And in our study, rosiglitazone increased the hepatic lipid accumulation. These results demonstrated that PPARγ2 plays an important role in hepatic lipid accumulation in NAFLD. Moreover, celastrol decreased the fatty acid binding protein 3 (Fabp3) and fatty acid transport protein 1 (Fatp1) levels (Figure 2D). By suppressing the expression of genes related to lipid transport, celastrol could hinder the absorption of lipids in the intestinal tract. Intracellular fatty acid transport is crucially influenced by Fabp3 and Fatp1. In this case, celastrol could potentially hinder the absorption of lipids in the intestinal tract by suppressing the transcriptional activity of PPARγ, which in turn inhibits the transport of lipids. Interestingly, significant differences, both of which decreased, were observed in the expression levels of several de novo fatty acid synthesis and lipid oxidative genes (Figure 2E,F). We speculated that celastrol might regulate lipogenic and lipid transport to ameliorate hepatic steatosis in cluster of differentiation 36 (*CD36*), medium chain acyl-CoA dehydrogenase (MCAD), acyl-CoA oxidase (*ACOX*), stearyl-CoA desaturase-1 (*SCD1*), fatty acid desaturase 2 (*Fads2*), acetyl-CoA carboxylase1 (*Acc1*), and fatty acid synthase (*Fasn*).

### 3.3. Rosiglitazone Can Reverse the Fatty Liver Protective Effects of Celastrol on Fatty Liver Tissue

**Figure 3:**
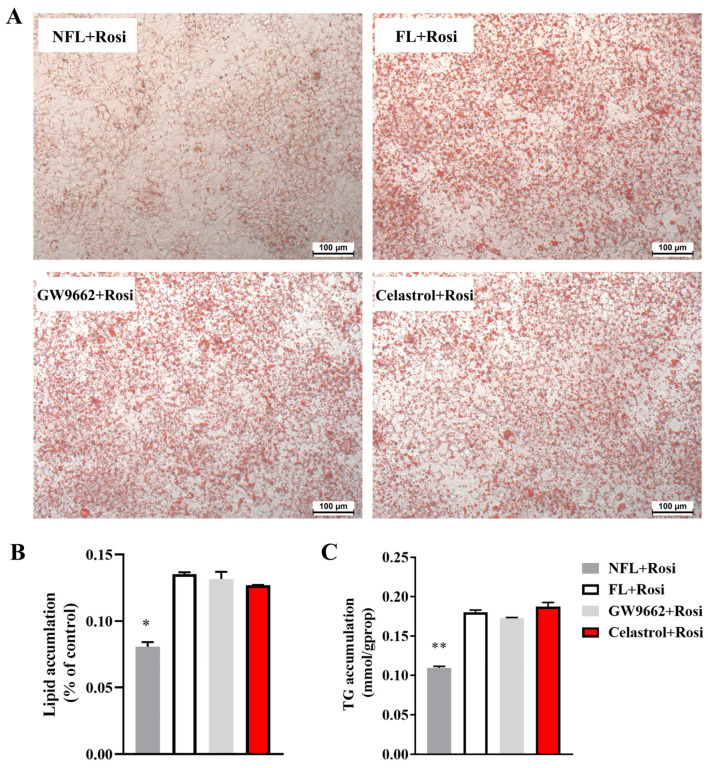
Celastrol can bind PPARγ, and rosiglitazone can reverse the protective effects of PPARγ inhibitors on fatty hepatocytes. OA-induced hepatic steatosis in HepG2 cells was treated with 1 µM rosiglitazone and 0.5 µM celastrol or 5 µM GW9662 for 48 h, and lipid accumulation was measured by (**A**) ORO staining, (**B**) quantified by biochemical analysis, and (**C**) densitometry analysis. The values are presented as the means ± SEMs; n = 3; * *p* < 0.05, ** *p* < 0.01 vs. the FL group. OA: oleic acid; ORO: Oil Red O; NFL: nonfatty liver group; FL: fatty liver group; Rosi: rosiglitazone.

Our study indicated that celastrol could directly bind to PPARγ and inhibit its transcriptional activity. Rosiglitazone acts as a full activator of PPARγ and can activate its transcriptional activity. We treated NAFLD cells with celastrol and rosiglitazone in combination with OA, and the results are shown in Figure 3. Celastrol improved OA-induced fatty tissue in HepG2 cells (Figure 1A,C,D), and rosiglitazone reversed the protective effects of celastrol on hepatic steatosis (Figure 3). The protective effect of GW9662, a PPARγ inhibitor, on fatty hepatocytes was also reversed by rosiglitazone (Figure 3).

### 3.4. Celastrol Does Not Influence Glucose Uptake Function

**Figure 4:**
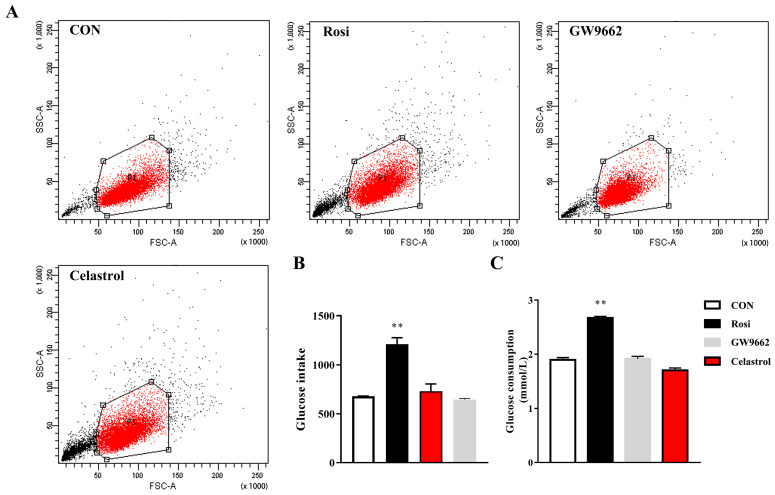
Glucose intake and consumption of HepG2 cells. (**A**,**B**) Glucose uptake analysis by flow cytometry. (**C**) Glucose consumption. The values are presented as the means ± SEMs; n = 3; ** *p* < 0.01 vs. the CON group. CON: untreated HepG2 cells; Rosi: rosiglitazone.

**Figure 5:**
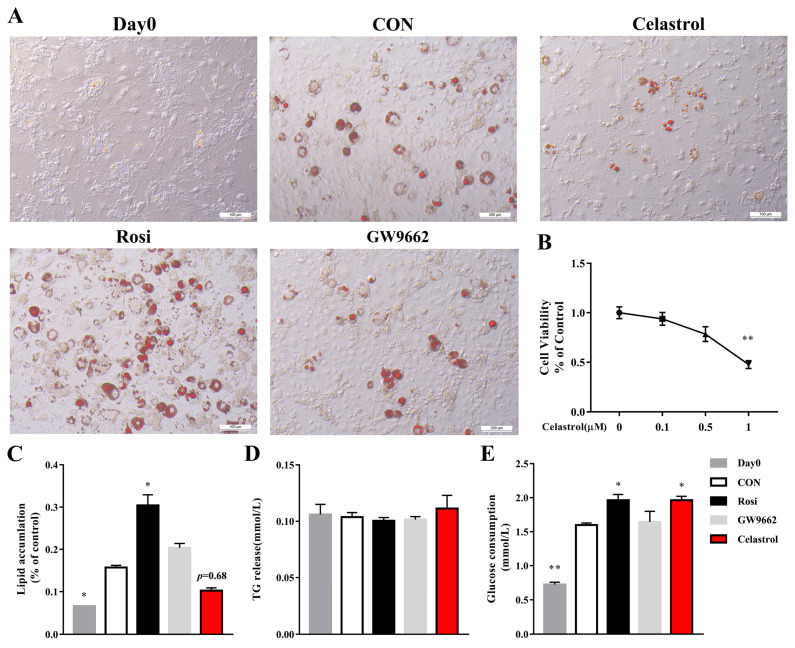
Celastrol inhibits adipocyte differentiation and increases glucose consumption in 3T3-L1 adipocytes. 3T3-L1 adipocytes were treated with celastrol at 0.5 µM for 48 h and lipid accumulation was measured via (**A**) ORO staining and (**C**) quantified by densitometry analysis. (**B**) Viability of 3T3-L1 adipocytes treated with celastrol at concentrations of 0, 0.1, 0.5, and 1 µM for 48 h. (**D**) TG release and (**E**) glucose consumption of 3T3-L1 adipocytes. The values are presented as the means ± SEMs; n = 3; * *p* < 0.05, ** *p* < 0.01 vs. the CON group. Day 0: 3T3-L1 preadipocytes; CON: untreated 3T3-L1 adipocytes; Rosi: rosiglitazone.

Rosiglitazone, a classical PPARγ agonist, acts primarily as effective insulin sensitizers, thereby reducing blood glucose levels [20,21]. Can celastrol affect glucose uptake function by inhibiting PPARγ. For HepG2 cells, rosiglitazone markedly increased glucose uptake in HepG2 cells, but celastrol and GW9662 did not influence the glucose uptake function (Figure 4A,B). The glucose consumption experiment also confirmed this finding (Figure 4C). Compared with the control, 3T3-L1 adipocytes can increase the glucose uptake (Figure 5D). In parallel, compared with the control, rosiglitazone and celastrol can increase the glucose consumption of 3T3-L1 adipocytes, while GW9662 barely changes the sugar absorption (Figure 5D).

### 3.5. Celastrol Inhibits Adipocyte Differentiation of 3T3-L1 Adipocytes

Obesity is considered the most vital common risk factor for developing NAFLD. In addition, in many conditions, the alleviation of obesity also ameliorates NAFLD. 3T3-L1 adipocytes have been widely used in anti-obesity studies to study important molecular markers of adipocyte differentiation [22]. 3T3-L1 preadipocytes were treated with various concentrations of celastrol (0, 0.1, 0.5, 1 µM), and cell viability was measured via a CCK8 assay. According to Figure 5B, the findings indicated that cell viability was not affected by 0.1 µM or 0.5 µM celastrol, demonstrating no evidence of toxicity. Thus, we tested the effect of a nontoxic concentration of 0.5 µM of celastrol on adipocyte differentiation.

To test the impact of celastrol on the adipocyte differentiation, 3T3-L1 adipocytes were subjected to celastrol, rosiglitazone, and GW9662 treatment for a duration of 48 h. The results indicated that the administration of celastrol effectively hindered the buildup of lipids in ORO-stained lipid droplets within 3T3-L1 adipocytes (Figure 5A,B). Then, we detected the TG release in drug-treated 3T3-L1 adipocytes. We found that no differences were demonstrated between the groups (Figure 5C). These results are consistent with the literature [7].

### 3.6. Celastrol Alters the Gene Expression Profile Associated with Adipocyte Differentiation in 3T3-L1 Adipocytes

**Figure 6:**
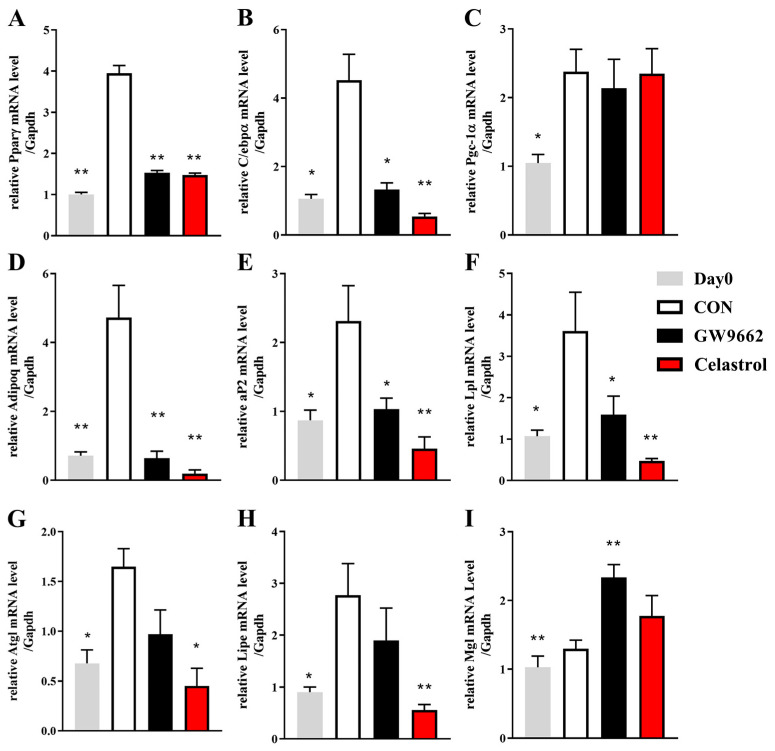
Celastrol inhibits the transcriptional activity of PPARγ. The adipocyte differentiation of 3T3-L1 adipocytes was treated with 5 μM GW9662 and 0.5 µM celastrol for 48 h, and the mRNA levels of (**A**–**I**) PPARγ, C/ebpα, Pgc-1α, Adipoq, aP2, Lpl, Atgl, Lipe, and Mgl were measured. The values are presented as the means ± SEMs; n = 3; * *p* < 0.05, ** *p* < 0.01 vs. the CON group. Day 0: 3T3-L1 preadipocytes; CON: untreated 3T3-L1 adipocytes.

PPARγ is the key process that promotes adipogenesis differentiation via controlling many downstream lipogenic genes. C/EBPα is a major transcription factor involved in lipid synthesis and is highly upregulated during the initial stages of preadipocyte maturation. To investigate adipocyte differentiation markers, we measured the mRNA levels of *PPARγ*, *C/EBPα*, fatty acid binding protein 4 (*aP2*), and adiponectin *(Adipoq*). The mRNA levels of 3T3-L1 preadipocytes were significantly upregulated during differentiation into mature adipocytes. Celastrol could work better at decreasing the expression of adipocyte differentiation marker genes than the other inhibitor GW9662 (Figure 6A–E). Celastrol treatment improved differentiation via the suppression of adipogenesis in 3T3-L1 adipocytes.

In the process of lipolysis, TGs undergo sequential reactions involving adipocyte triglyceride lipase (*Atgl*), hormone-sensitive lipase (*Lipe*), and monoglyceride lipase (*Mgl)* in the lipolysis process [23]. To evaluate the key adipocyte lipolysis markers, we identified the lipolysis signature genes, such as *Atgl*, *Lipe,* and *Mgl*. We observed a significant reduction in *Atgl* and *Lipe* in response to celastrol (Figure 6G,H) and a slight increase in *Mgl* (Figure 6I). *Mgl* is the last enzyme of the lipolysis pathway. Additionally, lipoprotein lipase (*LPL*), a negative regulator of lipolysis, controls the influx of free fatty acids (FFAs) into adipocytes [24]. Celastrol decreased the *LPL* mRNA level (Figure 6F).

## 4. Discussion

NAFLD is a widespread long-term liver condition worldwide. In recent years, the term MAFLD has been used instead of NAFLD. This is because NAFLD is usually accompanied by obesity, type 2 diabetes, and others related to disorders of glycolipid metabolism [25,26]. Currently, with a global incidence rate of 25%, NAFLD poses a significant burden on society. The increasing prevalence of NAFLD is also partially responsible for the rapid growth in hepatocellular carcinoma cases [27]. Hence, the management and prevention of NAFLD/MAFLD hold paramount importance in the field of public health. Over the past few years, several natural plant compounds, such as emodin [28], berberine [29], quercetin [30], and resveratrol [31], have demonstrated advantageous properties in inhibiting the development and progression of NAFLD by enhancing the dysfunction of hepatic glycolipid metabolism. Celastrol has been shown to ameliorate NAFLD and obesity [13,15,16]. Celastrol can stabilize glycolipid metabolism to improve NAFLD [16,17,32]. Celastrol has been found to potentially control lipid production by altering the phosphorylation of AMPKα, thereby decreasing the expression of SREBP-1c [33]. Choi et al. [7] demonstrated that treatment with celastrol inhibited adipocyte differentiation and increased lipolysis via the PPARγ2 and C/EBPα signaling pathways in 3T3-L1 adipocytes. PPARγ is mainly involved in adipogenesis, lipid metabolism, and glucose homeostasis [19]. Adenoviral PPARγ2 injection induces the overexpression of PPARγ2 and its downstream target gene CD36, which results in higher hepatic TG levels and higher liver weights than those of control mice [11]. PPARγ knockout mice presented lower hepatic TG levels and fewer lipid droplets than did the controls, but the body or liver weights did not change. The PPARγ-mediated adjustment of the balance of glycolipid metabolism and the improvement in insulin resistance may be key measures for the prevention and treatment of NAFLD with celastrol. The aim of this research was to investigate the in vitro impact of celastrol on reducing lipid accumulation through the PPARγ signaling pathway.

Take together, the findings of our research indicated that GW9662, a PPARγ inhibitor, and celastrol ameliorate OA-induced hepatic steatosis and that rosiglitazone exacerbates lipid accumulation. PPARγ is a core factor that regulates fatty acid metabolism and adipogenesis. Numerous studies have indicated that celastrol regulates the PPARγ signaling pathway [7,17,34]. Using a ligand binding assay based on SPR technology, we successfully demonstrated the binding of celastrol to PPARγ. An increase in the expression of PPARγ2 was observed in NAFLD patients [10]. PPARγ overexpression has been found to mitigate the effect on liver steatosis, a condition that can be alleviated by the administration of GW9662 [11]. In the present study, celastrol was shown to reduce the mRNA levels of PPARγ2 and PPARα, and GW9662 decreased the mRNA levels of PPARγ1 and 2. Additionally, rosiglitazone increased the mRNA level of PPARγ2. Rosiglitazone reversed the protective effect of celastrol treatment on NAFLD cells. These results might indicate that PPARγ2 is a crucial regulator in NAFLD progression.

PPARγ regulates lipid-metabolism-related downstream genes, including those involved in fatty acid transport, lipolysis, and adipogenesis. Also, we demonstrated that celastrol inhibited the transcriptional activity of PPARγ, thereby decreasing the *Fabp3*, *Fatp1*, *MCAD*, *Fads2*, and *ACC1* mRNA expression in OA-induced hepatic steatosis cells. Long-chain fatty acids are bound to FABP, FATP, and FAT, which are now considered competitive receptor-mediated transporters, then are taken into the hepatocytes [35]. PPARγ triggers the activation of downstream target genes such as *Fasn*, *Acc1*, *SCD1*, *Fads2*, and additional crucial enzymes engaged in lipid synthesis, thereby facilitating the synthesis of TG and the deposition of lipids. The dysregulation of *Fasn*, *Acc1*, and *Scd1* is observed in the livers of rats suffering from fatty liver and obesity [36]. Our results indicated that celastrol might reduce the uptake of oleic acid and long-chain fatty acid biosynthesis by decreasing *Fabp3*, *Fatp1*, *MCAD*, *Fads2*, and *Acc1* mRNA levels. Moreover, GW9662 might decrease fatty acid uptake by decreasing *Fabp3* and *Fatp1* mRNA levels, and it might decrease the long-chain fatty acid composition by decreasing *Fads2* mRNA levels.

NAFLD is closely related to obesity, but the relationship between obesity and NAFLD has not been determined. According to Choi [7], celastrol hindered the differentiation of adipocytes in 3T3-L1 cells by reducing glyceraldehyde-3-phosphate dehydrogenase (GPDH) activity and suppressing the transcriptional activity of PPARγ2. Different durations of celastrol treatment can induce different inhibitory effects in 3T3-L1 adipocytes, and differences in adipogenesis are shown in the PPARγ2 expression level. Additionally, PPARγ2 decreases in the late stages of differentiation. And C/ebpβ is induced by DEX and IBMX during adipocyte differentiation and can induce PPARγ2 and C/ebpα to activate the diverse adipocyte markers [37]. This might indicate that PPARγ is abundant in the middle term of adipocyte differentiation. In our research, celastrol was used on the 2nd–4th (the middle term) day of adipogenesis. Celastrol, which serves as an inhibitor of PPARγ, inhibits lipogenesis by decreasing PPARγ expression and transcriptional activity in 3T3-L1 adipocytes. Celastrol decreases the mRNA levels of aP2 and Adipoq in 3T3-L1 adipocytes. Studies have demonstrated that medications blocking aP2 and Adipoq hold promise for the treatment of obesity, diabetes, and NAFLD [38]. By enhancing leptin sensitivity, celastrol can suppress food consumption, block the reduction in energy expenditure, and result in a weight loss of up to 45% in hyperleptinemic-diet-induced obese mice [13]. Notably, this effect was not observed in the leptin-deficient (ob/ob) mouse model [13]. The Atgl/Lipe/Mgl axis is the major triglyceride hydrolase responsible for adipose lipolysis. The regulation of energy balance relies on leptin, which can enhance lipolysis by increasing the expression of Lipe, Atgl, and Mgl [39]. Celastrol decreases the mRNA levels of Atgl and Lipe in 3T3-L1 adipocytes. These findings might indicate that Atgl and Mgl are regulated by PPARγ. However, the underlying mechanisms linking the lipolysis gene and PPARγ require further exploration. During HFD feeding, neuron-specific PPARγ knockout mice exhibit a reduced weight gain via reduced food consumption and increased energy expenditure [40]. This observation suggested that the inhibitory effect of celastrol on appetite may be attributed to its inhibition of brain PPARγ.

The glucose uptake of untreated HepG2 cells remained unchanged by celastrol; however, celastrol enhanced glucose absorption in 3T3-L1 adipocytes. These findings are in line with what has been reported in the literature [13]. Studies have shown that regular-chow-diet-fed mice do not experience lipoatrophy or glucose intolerance despite the absence of PPARγ [11,41]. Interestingly, it offered defense against obesity and glucose tolerance in mice that were given a high-fat diet [42]. However, future studies are needed to further explore the potential underlying mechanisms involved. Through this investigation, we have discovered that celastrol has an affinity for and actively suppresses the transcriptional function of PPARγ. Celastrol can potentially alleviate hepatic steatosis induced by OA and hinder adipocyte differentiation, but it does not affect glucose uptake. This difference might be due to the different inhibitory effects of celastrol on PPARγ1 and PPARγ2. However, it is necessary to clarify the specific functions of the γ1 and γ2 isoforms in the coming years.

Furthermore, our investigation revealed that celastrol induced a modest level of cytotoxicity in HepG2 cells and 3T3-L1 adipocytes when celastrol was administered at elevated concentrations. Consequently, the imperative pursuit of modifying the structure of celastrol to mitigate its toxicity while preserving its therapeutic efficacy has emerged as a crucial point for future research. Since we did not look at the whole signaling pathway, additional work needs to be carried out in the future. In-depth in vivo studies could further elucidate the role of celastrol as a potential therapeutic agent for NAFLD/MAFLD.

## 5. Conclusions

To summarize, our findings indicate that celastrol can bind to and directly modulate the function of PPARγ. This leads to improvements in hepatic steatosis caused by oleic acid as well as adipocyte differentiation, as well as an increase in glucose uptake in 3T3-L1 adipocytes. Given that we did not perform experiments on the in vivo signaling pathway, there is a significant amount of additional research that must be conducted in the future. Despite these limitations, numerous other studies have provided evidence of the therapeutic effect of celastrol in NAFLD, obesity, and diabetes. The current experimental results validate the therapeutic impact of celastrol and offer a scientific justification for exploiting this compound for the treatment of hepatic steatosis reduction.

## Figures and Tables

**Table 1:** Primer sequences used in this study.

Primer Name	Primer Sequence (5′-3′)
H-PPARγ	Forward: GACCACTCCCACTCCTTTGA Reverse: CGACATTCAATTGCCATGAG
H-PPARγ1	Forward: AGGCGAGGGCGATCTTGACAG Reverse: GATGCGGATGGCCACCTCTTT
H-PPARγ2	Forward: GCCTTGCAGTGGGGATGTCTC Reverse: CCTGGGCGGTTGATTTGTCTG
H-PPARα	Forward: CTATCATTTGCTGTGGAGATCG Reverse: AAGATATCGTCCGGGTGGTT
H-CD36	Forward: CATCGCTGGGGCTGTCATT Reverse: GCGTCCTGGGTTACATTTTCC
H-Fabp3	Forward: TTCTGGAAGCTAGTGGACAG Reverse: TGATGGTAGTAGGCTTGGTCAT
H-Fatp1	Forward: TGACAGTCGTCCTCCGCAAGAA Reverse: CTTCAGCAGGTAGCGGCAGATC
H-MCAD	Forward: TAATCGGTGAAGGAGCAGGTTT Reverse: GGCATACTTCGTGGCTTCGT
H-ACOX	Forward: GTTTGGACTCCGCCACTGCTTG Reverse: GGCTGAACTCTGGCATCCACAT
H-SCD1	Forward: CAGGTTTCCAAGCGCAGTTC Reverse: ACTGGAGATCTCTTGGAGCA
H-Fads2	Forward: TTCCTGGAGAGCCACTGGTTTG Reverse: GAAGAAGGACTGCTCCACATTGC
H-Acc1	Forward: ATGGGCGGAATGGTCTCTTTC Reverse: TGGGGACCTTGTCTTCATCAT
H-Fasn	Forward: CACAGGGACAACCTGGAGTT Reverse: ACTCCACAGGTGGGAACAAG
H-Gapdh	Forward: ATGGATGATGATATCGCCGCC Reverse: CTCCATGTCGTCCAGTTGGT
M-PPARγ	Forward: TTTTCAAGGGTGCCAGTTTC Reverse: AATCCTTGGCCCTCTGAGAT
M-C/ebpα	Forward: CAAGAACAGCAACGAGTACCG Reverse: GTCACTGGTCAACTCCAGCAC
M-aP2	Forward: AGGTGAAGAGCATCATAACCCT Reverse: TCACGCCTTTCATAACACATTCC
M-Adipoq	Forward: TGTTCCTCTTAATCCTGCCCA Reverse: CCAACCTGCACAAGTTCCCTT
M-LPL	Forward: GGGAGTTTGGCTCCAGAGTTT Reverse: TGTGTCTTCAGGGGTCCTTAG
M-Pgc-1α	Forward: ACCCACAGGATCAGAACAAACCCT Reverse: TTGGTGTGAGGAGGGTCATCGTTT
M-Atgl	Forward: GTTGAAGGAGGGATGCAGAG Reverse: GCCACTCACATCTACGGAGC
M-Lipe	Forward: TCTCGTTGCGTTTGTAGTGC Reverse: ACGCTACACAAAGGCTGCTT
M-Mgl	Forward: AACCACTAAGCCCCAGTTCC Reverse: GCAGGATGTGAGCAGAGCAC
M-Gapdh	Forward: AACTTTGGCATTGTGGAAGG Reverse: ACACATTGGGGGTAGGAACAM

## Data Availability

The data presented in this study are available on request from the corresponding author. Data are not publicly available, due to privacy.

## Supplementary Materials

The following supporting information can be downloaded at: https://www.mdpi.com/article/10.3390/metabo14010064/s1, Figure S1: Different concentrations of oleic acid treatment in HepG2 cells.
