# Supplementary material for: Celastrol Stabilizes Glycolipid Metabolism in Hepatic Steatosis by Binding and Regulating the Peroxisome Proliferator-Activated Receptor γ Signaling Pathway
Source: Metabolites. 2024 Jan 19;14(1):64. doi: 10.3390/metabo14010064 (PMC10818689; doi:10.3390/metabo14010064)
Supplement: Supplementary file 1 [file metabolites-14-00064-s001.zip › metabolites-2768737-supplementary.pdf]

HepG2 cells were treated with oleic acid (OA) at concentrations of 0, 250, 500, 1000 μM for 48hous to establish non-alcoholic fatty liver disease cell model, after that, OA-treated HepG2 cells tested via a cell viability assay and oil red staining. These results were shown below. The cell viability only induced by 500μM OA was not affected and still be induced obvious lipid accumulation. Hepatic steatosis was unable to be induced by 250 μM OA, and 1000 μM OA treatment could produce a certain cytotoxicity. Hence, according to a comprehensive analysis of the results of oil red staining and cell viability assay, we finally used 500μM oleic acid for modeling under different concentration of 250, 500 and 1000μM oleic acid treatment. And these results will be presented in supplementary documents.

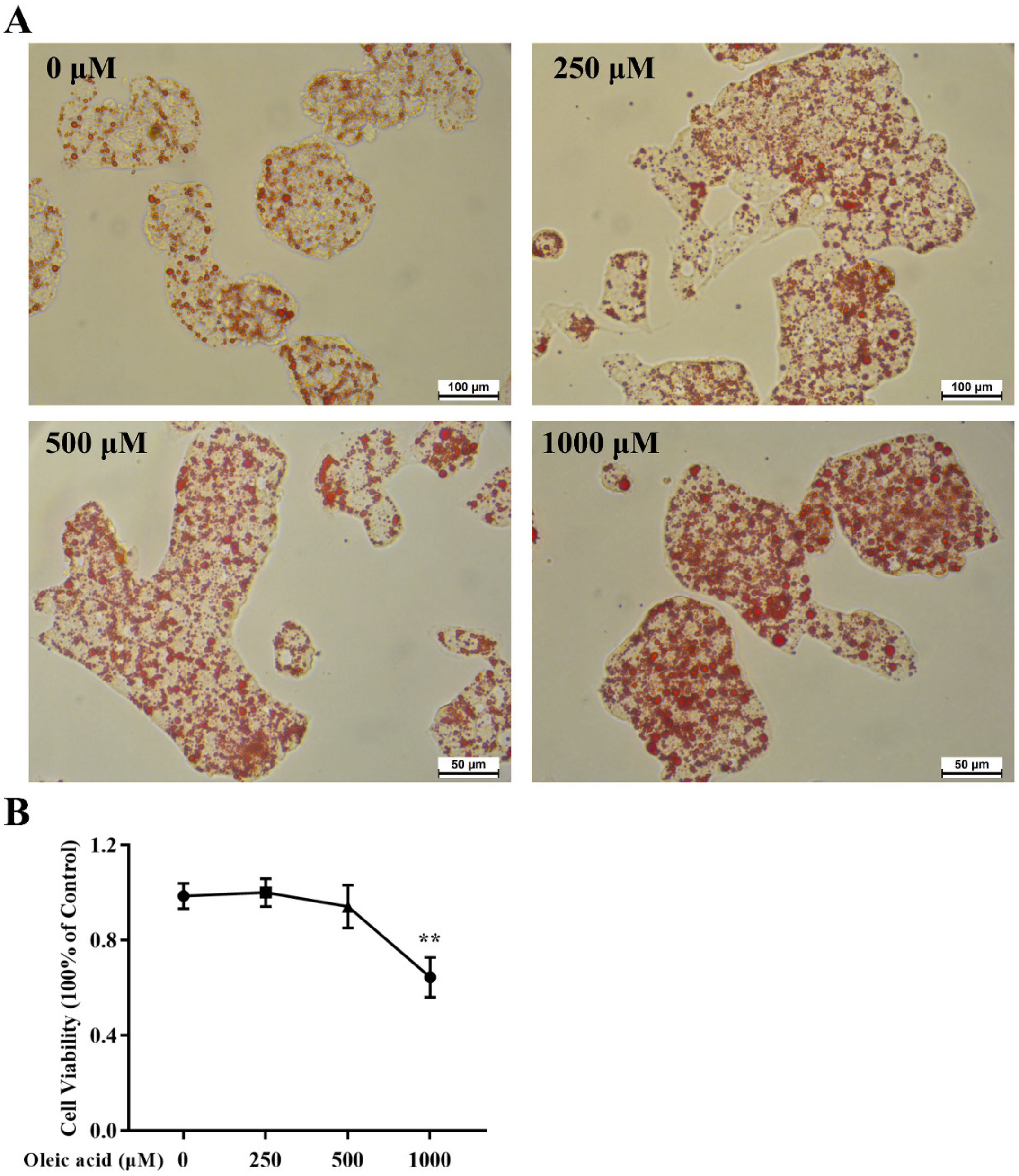

Supplementary Figure S1. Different concentrations of oleic acid treatment in HepG2 cells. (A) Oil red staining and (B) the cell viability assay at different concentrations of oleic acid treatment in HepG2 cells. ** $p < 0.01$ vs. the 0 μM oleic acid group.
